# Antioxidant Effects of Argan Oil and Olive Oil against Iron-Induced Oxidative Stress: In Vivo and In Vitro Approaches

**DOI:** 10.3390/molecules28155924

**Published:** 2023-08-07

**Authors:** Habiba Bouchab, Soukaina Essadek, Soufiane El Kamouni, Khadija Moustaid, Abdelkhalid Essamadi, Pierre Andreoletti, Mustapha Cherkaoui-Malki, Riad El Kebbaj, Boubker Nasser

**Affiliations:** 1 Laboratory of Biochemistry, Neurosciences, Natural Resources and Environment, Faculty of Sciences and Technologies, Hassan First University, Settat 26000, Morocco; habibabouchab78@gmail.com (H.B.); ess.soukaina@hotmail.fr (S.E.); eks.soufiane@gmail.com (S.E.K.); essamadi@uhp.ac.ma (A.E.); 2 Laboratory of Health Sciences and Technologies, Higher Institute of Health Sciences, Hassan First University, Settat 26000, Morocco; 3 Bio-PeroxIL Laboratory, EA7270, Université de Bourgogne, 6 Boulevard Gabriel, 21000 Dijon, France; pierre.andreoletti@u-bourgogne.fr (P.A.); mustapha.cherkaoui-malki@u-bourgogne.fr (M.C.-M.); 4 Laboratory of Applied Chemistry and Environment, Faculty of Sciences and Technologies, Hassan First University, Settat 26000, Morocco; khadija.moustaid@uhp.ac.ma

**Keywords:** oxidative stress, antioxidant enzymes, ferrous sulfate, argan oil, olive oil, brain, liver, kidney, *Tetrahymena pyriformis*

## Abstract

Recently, the study of the protective powers of medicinal plants has become the focus of several studies. Attention has been focused on the identification of new molecules with antioxidant and chelating properties to counter reactive oxygen species (ROS) involved as key elements in several pathologies. Considerable attention is given to argan oil (AO) and olive oil (OO) due to their particular composition and preventive properties. Our study aimed to determine the content of AO and OO on phenolic compounds, chlorophylls, and carotenoid pigments and their antioxidant potential by FRAP and DPPH tests. Thus, several metallic elements can induce oxidative stress, as a consequence of the formation of ROS. Iron is one of these metal ions, which participates in the generation of free radicals, especially OH from H_2_O_2_ via the Fenton reaction, initiating oxidative stress. To study the antioxidant potential of AO and OO, we evaluated their preventives effects against oxidative stress induced by ferrous sulfate (FeSO_4_) in the protozoan *Tetrahymena pyriformis* and mice. Then, we evaluated the activities of the enzymatic (superoxide dismutase (SOD), glutathione peroxidase (GPx)) and metabolite markers (lipid peroxidation (MDA) and glutathione (GSH)) of the antioxidant balance. The results of the antioxidant compounds show that both oils contain phenolic compounds and pigments. Moreover, AO and OO exhibit antioxidant potential across FRAP and DPPH assays. On the other hand, the results in *Tetrahymena pyriformis* and mice show a variation in the level of iron-changed SOD and GPx activities and MDA and GSH levels. By contrast, treating *Tetrahymena pyriformis* and mice with argan and olive oils shows significant prevention in the SOD and GPx activities. These results reveal that the iron-changed ROS imbalance can be counteracted by AO and OO, which is probably related to their composition, especially their high content of polyphenols, sterols, and tocopherols, which is underlined by their antioxidant activities.

## 1. Introduction

Redox balances and maintaining their homeostasis are fundamental to biological systems and very important for cell survival, which involves free radicals as reactive oxygen species (ROS). ROS are routinely derived from normal cellular oxidative metabolism [1], including hydroxyl radicals (OH), superoxide anion (O^2−^), and hydrogen peroxide (H_2_O_2_) [2]. At a low level of production, ROS are signal molecules that regulate a wide range of physiological processes [3], and play diverse roles in cell growth, survival, and proliferation [4]. However, when formed at a high level, ROS trigger oxidative stress in living organisms [5]. To defend against oxidative stress, cells have evolved various enzymatic and non-enzymatic antioxidant defense systems. The primary enzymatic antioxidant includes catalase (CAT), glutathione peroxidase (GPx), and superoxide dismutase (SOD), while the second line of defense includes antioxidants such as GSH [6].

Accordingly, several metal elements can induce oxidative stress, as a consequence of the formation of ROS [7]. Iron, as one of these metal ions, is an essential constituent of the human body and a key player in oxygen transport and enzymatic functions [8]. Besides this, it participates also in the generation of free radicals [9], especially OH from H_2_O_2_ via the Fenton reaction, initiating oxidative stress [10]. Such oxidative stress is involved in the deterioration of cell constituents such as proteins, nucleic acids, and lipids [11], and is strongly implicated in the genesis of several neurodegenerative diseases such as Parkinson’s disease (PD), amyotrophic lateral sclerosis (ALS), Huntington’s disease (HD), and Alzheimer’s disease [12]. Nevertheless, various existing cell self-defense mechanisms counteract this oxidative stress, including catalase (CAT), glutathione peroxidase (GPx), superoxide dismutase (SOD), and malondialdehyde (MDA) [6]. Numerous iron chelators have been chemically synthesized to treat many diseases related to iron overload [13] such as deferasirox (DFX), deferoxamine (DFO), and deferiprone (DFP). However, these drugs have limitations due to their side effects during acute and chronic administration [14], prompting many researchers to identify new molecules from medicinal plants, already used in traditional medicine pharmacopeia, with antioxidant and/or chelating iron activities. In this context, we previously focused our studies on the biological activities of vegetable oils. Hence, the consumption of certain oils known for their beneficial effects on health such as argan oil (AO), used in Morocco, and olive oil (OO), a usual ingredient in the Mediterranean diet, could have also beneficial effects on oxidative stress by stabilizing the activities of stress enzymes and inflammation [15,16,17,18]. In addition, argan oil obtained from the *Argania spinosa* tree has been used for centuries in the traditional Moroccan Amazigh food [19], while OO is well known as a Mediterranean diet component [20]. Chemical analyses show that AO and OO contain an unsaponifiable fraction, including tocopherols, polyphenols compounds, and phytosterols. AO is notable for high levels of schottenol and spinasterol as phytosterols, while OO predominantly contains β-sitosterol as its primary phytosterol compound [17,21]. A recent study reports that both oils have cytoprotective potential on 158N (A) and BV2 cells from 7-ketocholesterol toxicity [22]. It has been reported that phenolic compounds from medicinal plants play a role in preventing different diseases related to oxidative stress [23]. Interestingly, the AO saponifiable fraction contains 45% of the monounsaturated oleic acid (C18:1, Δ9) and 35% of the polyunsaturated linoleic acid (C18:2, Δ6), while the OO saponifiable fraction is composed of 75% oleic acid and 9% of linoleic acid, leading to a higher unsaturation index of 120.4 for AO versus 108.3 for OO [24]. Numerous studies revealed their anti-inflammatory, antioxidant, hepatoprotective, anti-DNA damage, and neuroprotective effects [16,18,25]. Therefore, it was interesting to specify its antioxidant activities and its protective effects on the toxicity related to iron overload. This work aimed to study the protective and antioxidant effect of AO and OO on induced oxidative stress by iron (FeSO_4_) overload in *Tetrahymena pyriformis* (*T. pyriformis*), a eukaryotic ciliated protozoan. This well-known unicellular model has been widely used by many researchers in physiological and toxicological studies [26]. Due to its characteristics, a protozoan combines the complexity of cellular eukaryotic functions and structures comparable to that found in human cells [27]. *Tetrahymena* is also used to understand the genetic system of higher organisms [28]. In the present study, the antioxidant potential of AO and OO was assessed by the determination of chlorophyll, carotenoids, and phenolic compound amounts as well as the evaluation of their antioxidant capacity by FRAP and DPPH assays. Indeed, we investigated the antioxidant effects of argan oil in iron-induced oxidative stress in vivo mice and in vitro in *Tetrahymena pyriformis*. Three organs were chosen in this present study: the liver, brain, and kidney, due to their high sensitivity to iron overload, which is known to disturb the intracellular redox balance and cause oxidative stress. The antioxidant potential of argan oil was evaluated through the measurement of two antioxidant enzymes glutathione peroxidase (GPx) and superoxide dismutase (SOD) and biomarkers such as malondialdehyde (MDA) and reduced glutathione (GSH). The effects of AO against the toxicity of iron are compared to those of olive oil (OO), a well-known component in the traditional Mediterranean diet.

## 2. Results

The data of the literature show that AO and OO are natural oils characterized by a particular composition with a predominance of unsaturated fatty acids and a fraction rich in antioxidants such as polyphenols, chlorophylls, and carotenoid pigments. To ensure the protective quality of AO and OO, we characterized the two oils and, subsequently, we evaluated their possible antioxidant activity.

### 2.1. Total Polyphenol Contents, Pigments Amounts, and Antioxidant Activities

**Figure 1:**
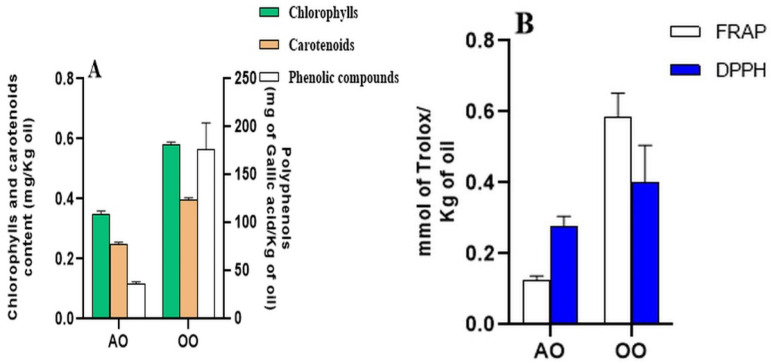
(**A**) Total phenolic content (TPC) (mg of Gallic acid per kilogram of oil), chlorophylls, and carotenoid content (mg/Kg oil). (**B**) Antioxidant activity (FRAP, DPPH) (mmol of TE/kg oil) determined in the argan oil (AO) and olive oil (OO) from Morocco (*n* = 3). TPC test uses gallic acid as a reference molecule. FRAP and DPPH tests use Trolox as a reference molecule.

Chlorophylls and carotenoids are the main pigments in vegetable oils. These pigments act as pro-oxidants in the presence of light and as antioxidants in the dark [29]. The results of the chlorophyll and carotenoid amounts (Figure 1A) show that the OO contains higher amounts, with 0.58 and 0.39 mg/kg, respectively, than the AO, with 0.34 and 0.24 mg/kg, respectively. The differentiation of oils based on their content in polyphenols, as well as on their antioxidant activities, is a complex task since these criteria depend on several parameters such as specie polymorphism, altitude, climate factors, temperature, rain, soil type, drought, harvest year, and the fruit maturity, in addition to the oil extraction methods [30,31]. In our study, the assessment of the total phenol content of two different vegetable oils used in the experiment (Figure 1A) shows that OO contains more polyphenols than AO with 175.911 and 36.237 mg GAE/kg oil, respectively.

To evaluate the possible antioxidant properties of vegetable argan and olive oils, two conventional methodologies were used: DPPH and FRAP assays (Figure 1B). According to the two tests, the antioxidant activities of OO show a higher antioxidant potential across the two tested assays of DPPH and FRAP, with 0.401 and 0.585, respectively, compared to AO with 0.275- and 0.123-mM TE/kg oil, respectively. The phenolic content of OO is 4.85-fold higher than that of AO, which is in correlation with the OO to AO ratio (relative to the antioxidant activities) calculated by FRAP assay (4.75 folds). However, the antioxidant potential of OO across the DPPH assay is just 1.46-fold higher than AO.

### 2.2. IC50 of Iron

**Figure 2:**
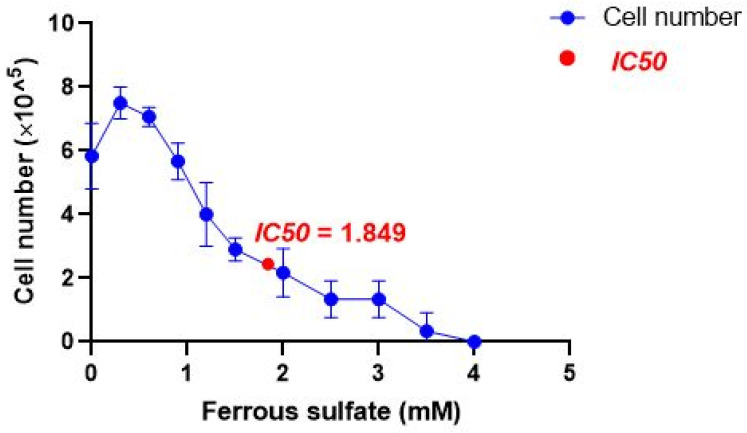
Effect of ferrous sulfate on *T. pyriformis*. The number of *T. pyriformis* cells was determined in the presence of different concentrations of iron. Values are given as mean ± SD. The 50% inhibitory concentration value (IC50) was carried out by Graph Pad Prism (*n* = 3).

*T. pyriformis* cell growth was followed in a stressful environment caused by FeSO_4_ used to generate oxidative stress in the protozoa with an increased iron concentration. The results of cell density of *T. pyriformis* shows that at low concentrations, below 0.5 mM, iron seems to stimulate the cell growth of *T. pyriformis* by nearly 30% (from 5.8 × 10^5^ without iron to 7.5 × 10^5^ cells per ml in the presence of 0.3 mM FeSO_4_), whereas iron concentrations of 0.5 mM to 4 mM negatively affect the *T. pyriformis* growth in a dose-dependent manner (Figure 2) up to complete cell growth inhibition. Therefore, iron at high concentrations could be an anti-growth agent because of its toxicity. In these conditions, the IC50 of FeSO_4_ was calculated a 1.85 mM to create the oxidative stress environment.

### 2.3. Argan Oil Protects T. pyriformis from Iron-Disturbed Antioxidant Activities

**Figure 3:**
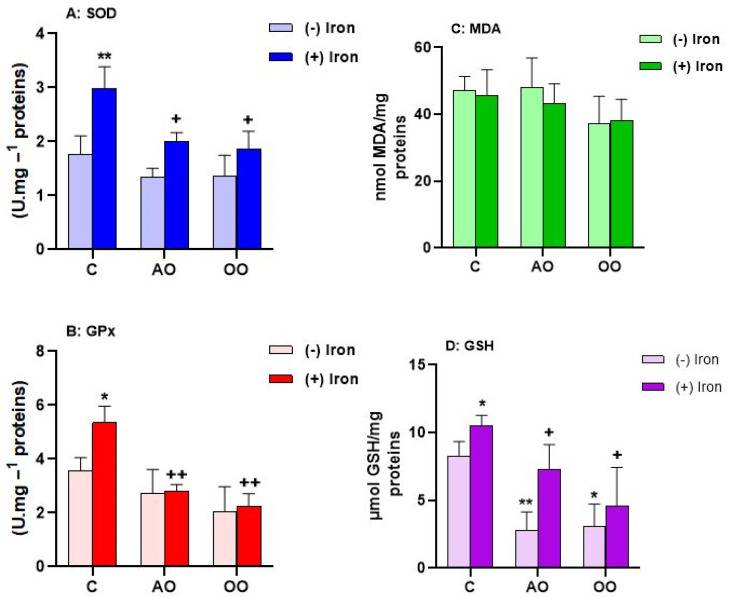
Antioxidant capacities of AO and OO in *Tetrahymena pyriformis* upon iron treatment. (**A**) Superoxide dismutase activity (SOD). (**B**) Glutathione peroxidase activity (GPx). (**C**) Lipid peroxidation level (MDA). (**D**) Glutathione level (GSH). After 24 h of culture in PPYG medium, cells without iron or oils (control: C); cells supplemented with 1849 µM of iron (FeSO_4_), 0.1% of argan oil (AO) or olive oil (OO), iron plus AO (FeSO_4_ + AO) or OO (FeSO_4_ + OO) were incubated for another 24 h. All values are means ± SD of triplicate analysis. Significant difference: ** *p* ˂ 0.01, * *p* ˂ 0.05 compared to the control and, ^++^ *p* ˂ 0.01, ^+^ *p* ˂ 0.05 compared to FeSO_4_. (*n* = 3).

To test the hypothesis on the potential protective effect of AO against iron-generated oxidative stress, we evaluated the antioxidant defense in *T. pyriformis*. Accordingly, antioxidant enzyme activities, including glutathione peroxidase (GPx) and superoxide dismutase (SOD), as well as oxidative stress markers, including glutathione and malondialdehyde levels, were analyzed after iron treatment in the presence or the absence of AO or OO pretreatment. As shown in Figure 3, the MDA level is not affected by iron treatment or by AO or OO. However, in the iron-treated cells, the GSH level increases by 27.17%. The combination of AO and iron or OO and iron shows a significant decrease in GSH level by 38.70% in AO and by 71.76% in OO-pretreated cells compared to iron treatment. Likewise, the iron treatment induces an increase in the SOD and GPx activities by 69.67% and 50.00%, respectively, when compared to the control untreated cells; this rise is countered by supplementation of AO and OO, which shows a return to control level in GPx activity (71.08% in AO and 86.65% in OO pretreated cells) and a decrease in SOD activity (56.62% in AO and 63.62% in OO pretreated cells), compared to the untreated cells. SOD activity is reduced by 56.61% in AO-treated cells and by 63.62% in OO-treated cells. Moreover, the AO and OO treatment has no differential effect on GPx, SOD, and MDA levels. However, these oils have a negative effect on the GSH marker. These results (Table 1) show that AO and OO prevent protozoan cells from changes in antioxidant capacities induced by iron treatment.

To confirm in vivo the antioxidant effect of AO demonstrated by the DDPH and FRAP tests, and also by the in vitro study in a unicellular *Tetrahymena* model, we used a pluricellular animal model (mouse). In addition, the antioxidant potential of AO using chemical assays was already explored. To date, articles on the preventive effect of AO on oxidative stress using the animal model in vivo are still lacking.

### 2.4. Argan Oil Protects the Liver from Iron-Disturbed Antioxidant Activities

**Figure 4:**
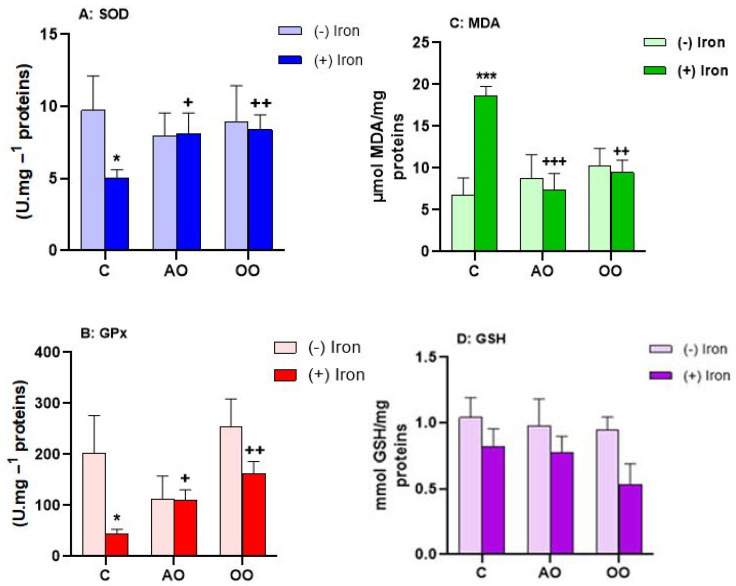
Antioxidant capacities of AO and OO in mouse liver upon iron treatment. (**A**) Superoxide dismutase activity (SOD). (**B**) Glutathione peroxidase activity (GPx). (**C**) Lipid peroxidation level (MDA). (**D**) Glutathione level (GSH). Mice received, for 28 days, a standard chow (control: C); a standard chow supplemented with 6% (*w*/*w*) argan oil (AO); a standard chow supplemented with 6% (*w*/*w*) olive oil (OO); a standard chow with the reference drug Tardyferon (iron sulfate 3.5 mg Fe^2+^/liter) dissolved in drinking water; the animals received iron and also AO; the animals received iron and also OO. All values are means ± SD (at least *n* = 3 per group). Statistical significance of higher mean signal intensity (*** *p* ˂ 0.001, * *p* ˂ 0.05) compared to control and (^+++^ *p* ˂ 0.001, ^++^ *p* ˂ 0.01, ^+^ *p* ˂ 0.05) compared to iron.

Iron overload is known to disrupt intracellular redox balance and cause oxidative stress. The antioxidant capacity of AO and OO was studied in the liver of experimental animals. As shown in Figure 4, AO and OO treatments have no differential effects on GPx, SOD activity, and MDA and GSH levels. However, iron treatment results in an increase in lipid peroxidation products (expressed in MDA equivalents), which is +176.67% higher than the control group, unlike GSH, which is not affected by iron treatment. Combinations of AO and iron or OO and iron result in the restoration of MDA expression to control levels. On the other hand, compared with group C, the hepatic SOD and GPx activities in the iron group are significantly decreased by −48.08% and −77.79%, respectively. Adding AO or OO along with iron treatment can counteract this effect, ensuring a return to the control level (Table 1).

### 2.5. Argan Oil Protects the Brain from Iron-Disturbed Antioxidant Activities

**Figure 5:**
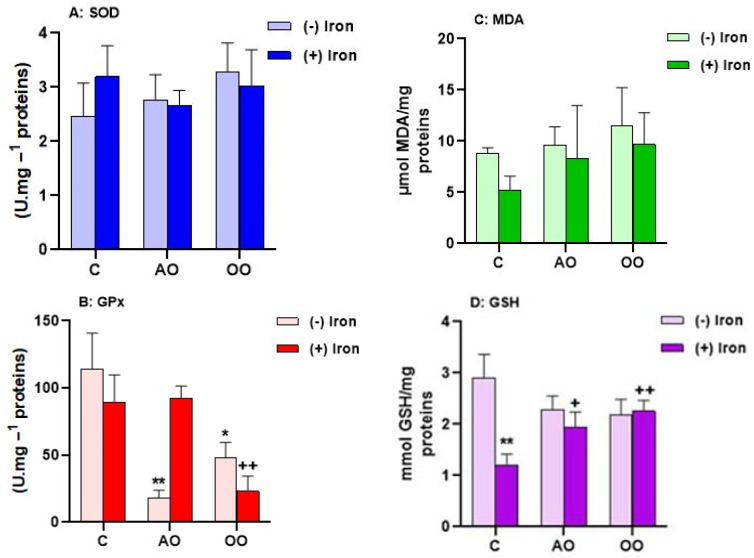
Antioxidant capacities of AO and OO in mouse brain upon iron treatment. (**A**) Superoxide dismutase activity (SOD). (**B**) Glutathione peroxidase activity (GPx). (**C**) Lipid peroxidation level (MDA). (**D**) Glutathione level (GSH). Mice received, for 28 days, a standard chow (control: C); a standard chow supplemented with 6% (*w/w*) argan oil (AO); a standard chow supplemented with 6% (*w/w*) olive oil (OO); a standard chow with the reference drug Tardyferon (iron sulfate 3.5 mg Fe^2+^/liter) dissolved in drinking water; the animals received iron and also AO; the animals received iron and also OO. All values are means ± SD (at least *n* = 3 per group). Statistical significance of higher mean signal intensity (** *p* ˂ 0.01, * *p* ˂ 0.05) compared to control and (^++^ *p* ˂ 0.01, ^+^ *p* ˂ 0.05) compared to iron.

The brain is one of the human body’s largest and most complex organs. The brain’s main function is to control the organism’s actions according to the sensory information that reaches it. The brain is the most sensitive tissue to oxidative stress because it produces a large amount of ROS due to its constant need for oxygen. Therefore, it is very important to see the brain’s response to argan oil treatment as well as iron-induced stress by measuring the activity of stress markers. In this direction, we investigated the effect of iron overload on oxidative stress by measuring the activity of antioxidant enzymes including glutathione peroxidase (GPx) and superoxide dismutase (SOD) in the mouse brain. The results (Figure 5) show that iron does not affect the two enzymes studied. Interestingly, treatment with either AO or OO shows a decrease in GPx activity; this decrease persists only after OO and iron treatment. On the other hand, assessment of markers of oxidative stress do not reveal the effect of different treatments on MDA levels. However, GSH levels are significantly reduced in iron-treated animals by −58.62% compared to C, and oil addition treatment results in significantly increased GSH levels in AO (+25.33%) and OO (+36.25%) compared with iron-overloaded mice (Table 1).

### 2.6. Argan Oil Protects the Kidneys from Iron-Disturbed Antioxidant Activities

**Figure 6:**
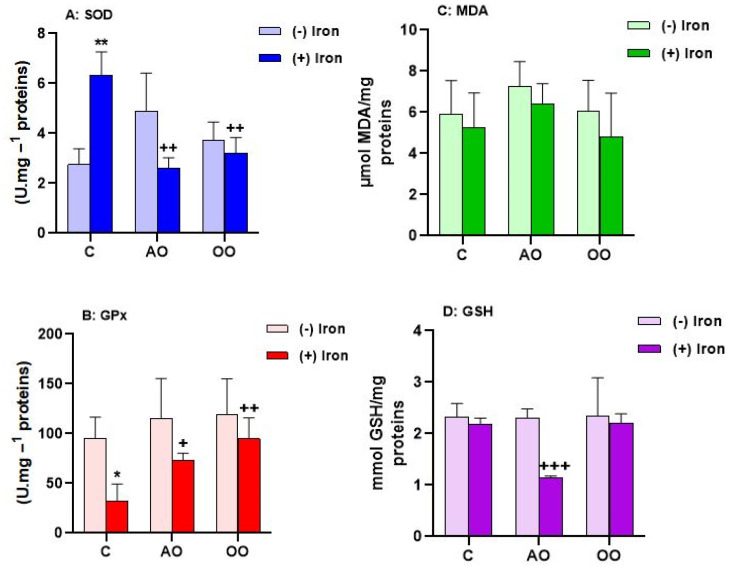
Antioxidant capacities of AO and OO in mouse kidney upon iron treatment. (**A**) Superoxide dismutase activity (SOD). (**B**) Glutathione peroxidase activity (GPx). (**C**) Lipid peroxidation level (MDA). (**D**) Glutathione level (GSH). Mice received, for 28 days, a standard chow (control: C); a standard chow supplemented with 6% (*w/w*) argan oil (AO); a standard chow supplemented with 6% (*w/w*) olive oil (OO); a standard chow with the reference drug Tardyferon (iron sulfate 3.5 mg Fe^2+^/liter) dissolved in drinking water; the animals received iron and also AO; the animals received iron and also OO. All values are means ± SD (at least *n* = 3 per group). Statistical significance of higher mean signal intensity (** *p* ˂ 0.01, * *p* ˂ 0.05) compared to control and (^+++^ *p* ˂ 0.001, ^++^ *p* ˂ 0.01, ^+^ *p* ˂ 0.05) compared to iron.

The kidneys provide several essential functions for the body. On the one hand, they make it possible to eliminate endogenous or exogenous waste. On the other hand, they play a role in maintaining homeostatic balance. Many studies show that ROS produced following iron overload are involved in the appearance of many kidney pathologies [32]. According to the determined results (Figure 6), a paradoxical variation of the GPx and SOD activity is highlighted. Overall, the iron treatment causes a significant decrease in GPx (−66.28%) and a very significant increase in SOD (+130.50%) is recorded compared to the control group. Iron-induced changes in GPx and SOD are strongly normalized by adding both oils to the diets for SOD and especially OO for GPx. On the other hand, the assessment of the stress oxidative marker shows that iron has no significant effect on renal MDA and GSH levels of any groups (Table 1).

**Table 1:** Summary table of the effect of argan and olive oil on the antioxidant capacities of the protozoan *Tetrahymena pyriformis* and the liver, kidney, and brain in mice following iron-induced toxicity. C: Control; AO: Argan oil and OO: Olive oil. All values are means ± SD (at least n = 3 per group). Statistical significance of higher mean signal intensity (*** *p* ˂ 0.001, ** *p* ˂ 0.01, * *p* ˂ 0.05) compared to control and (^+++^ *p* ˂ 0.001, ^++^ *p* ˂ 0.01, ^+^ *p* ˂ 0.05) compared to iron.

Sample		SOD	GPx	MDA	GSH
***Tetrahymena pyriformis***	**C**	1.763 ± 0.346	3.572 ± 0.478	47.154 ± 4.173	8.273 ± 1.068
	**Iron**	2.992 ± 0.393 **	5.358 ± 0.604 *	45.730 ± 7.603	10.521 ± 0.748 *
	**AO + Iron**	1.994 ± 0.173 ^+^	2.819 ± 0.239 ^++^	43.243 ± 5.873	7.319 ± 1.806 ^+^
	**OO + Iron**	1.870 ± 0.322 ^+^	2.263 ± 0.450 ^++^	38.285 ± 6.185	4.584 ± 2.845 ^+^
**Liver**	**C**	9.728 ± 2.391	202.959 ± 73.156	6.737 ± 2.081	1.040 ± 0.151
	**Iron**	5.051 ± 0.550 *	45.064 ± 8.087 *	18.638 ± 1.113 ***	0.817 ± 0.139
	**AO + Iron**	8.104 ± 1.448 ^+^	110.850 ± 19.796 ^+^	7.442 ± 1.930 ^+++^	0.772 ± 0.125
	**OO + Iron**	8.400 ± 1.015 ^++^	163.332 ± 22.959 ^++^	9.469 ± 1.487 ^++^	0.536 ± 0.156
**Brain**	**C**	2.471 ± 0.604	114.339 ± 26.456	8.801 ± 0.564	2.905 ± 0.455
	**Iron**	3.197 ± 0.570	89.673 ± 20.172	5.227 ± 1.364	1.202 ± 0.212 **
	**AO + Iron**	2.657 ± 0.284	92.365 ± 9.075	8.298 ± 5.173	1.938 ± 0.297 ^+^
	**OO + Iron**	3.027 ± 0.665	23.030 ± 11.234 ^++^	9.696 ± 5.173	2.255 ± 0.211 ^++^
**Kidney**	**C**	2.747 ± 0.634	95.221 ± 29.860	5.920 ± 1.620	2.318 ± 0.268
	**Iron**	6.333 ± 0.933 **	32.101 ± 18.016 *	5.242 ± 1.693	2.190 ± 0.112
	**AO + Iron**	2.624 ± 0.401 ^++^	72.961 ± 8.967 ^+^	6.390 ± 1.010	1.134 ± 0.042
	**OO + Iron**	3.216 ± 0.615 ^++^	94.682 ± 21.193 ^++^	4.820 ± 2.106	2.202 ± 0.185 ^+++^

## 3. Discussion

Argan and olive oils are two popular edible oils with unique compositions. AO has a balanced proportion of unsaturated fatty acids, including oleic acid (32.2%) and linoleic acid (46.4%), while OO is primarily composed of oleic acid (76.35%) with lower linoleic acid content (9.95%) [33]. Both oils contain antioxidant molecules in their unsaponifiable fraction, with AO having higher levels of γ-tocopherol and OO containing more α-tocopherol. AO also contains unique sterols such as schottenol and spinasterol [34], which are not found in OO [33]. Both oils have polyphenols with antioxidant potential [35] and contain minor compounds such as carotenoids and chlorophylls [36]. To ensure the protective quality of AO and OO, we characterized both oils and then evaluated their antioxidant potential. The oil characterization results show that OO is rich in chlorophylls and carotenoids compared to AO. Our results are consistent with the results in the literature that demonstrate the presence of carotenoid and chlorophyll pigments in AO and OO [36,37]. The variability in pigment contents could be explained by the influence of several factors such as roasting of seeds, temperature, time, extraction process, and storage conditions [36]. Lately, the antioxidant potential of vegetable oils has attracted great attention as an index of its quality. This potential is positively influenced by the presence of secondary metabolites, especially phytosterols, tocopherols, and polyphenols compounds [17], in addition to their fatty acids composition, especially oleic and linoleic acids [24]. The antioxidant activity is also known for the ability to chelate metals such as ferrous ions [31]. Phenols belong to secondary metabolites and are considered non-essential dietary components in plants [38]. They are present in all vegetable oils [39] and our results are in good agreement with these reports. The variability of amounts of polyphenols might vary depending on the degree of maturity [40], the storage time of the fruits before milling [41], the harvest period, the plant development stage, the genetic heritage [42], and the quantification method may also influence the estimation of phenol quantity. According to the literature, polyphenols, chlorophylls, and carotenoids have an antioxidant potential [25,43]. In recent decades, there has been a growing interest in studies of the antioxidant activity of food and diets due to the known implications of free radicals in the development of several diseases [44]. For this, we were interested in evaluating the antioxidant activity of AO and OO. The assessment of the reducing potential of iron by the FRAP test shows that OO has a much greater Fe^3+^ reducing activity compared to AO, up to 5.6 times higher. Almost the same potential was reported for Spanish extra virgin olive oil [45], which leads us to conclude that even by changing the origin of the oil, OO retains its antioxidant capacity. With regard to AO, similar results were found in the AO from the same origin as the oil tested [46]. This suggests that the iron-reducing potential of AO is not influenced by the period of harvest. Comparing the results of the polyphenol content with those of the test FRAP, there is a positive correlation as it is the same trend of variation. In addition, the results obtained from the DPPH test show that OO has a higher activity than AO, in the same range of values obtained by Samaniego and Marfil, respectively [47,48]. However, the DPPH results show that the polyphenols content of OO is 5 times higher than that of AO, and, intriguingly, its antioxidant power is only 1.5 times higher than AO, which could be explained by the main phenolic components of AO having the same antioxidant activity as the phenolic components of OO [49]. The two tests used in our study to measure antioxidant activity (FRAP and DPPH) provide preliminary information on the possible antioxidant effects of oils. The results obtained enable us to suggest that oils contain compounds endowed with antioxidant activities against free radicals and iron reduction. Many metals can induce oxidative stress through the formation of ROS. Iron is one of them. It is an essential component of living cells, maintaining human health. However, excess iron can lead to iron overload, which is associated with various diseases such as chronic liver injury [50]*,* because the liver is the organ responsible for iron storage and homeostasis [51]. In the current study, it is chosen as an oxidative stress agent because of its ability to form ROS through the Fenton and Haber–Weiss reaction [52], which, in turn, trigger oxidative damage to lipids, proteins, and DNA. We, therefore, tested the protection of oils against iron-induced oxidative stress in vitro (*Tetrahymena piriformis*) and in vivo (mice). The choice of Tetrahymena pyriformis protozoa in this study as a model organism has various advantages such as the fact it is a single-celled microscopic eukaryotic organism suitable for pharmacological experiments, allowing the use of common markers in animal experiments such as specific enzymes and growth. The toxicity of chemicals on Tetrahymena was evaluated based on the decrease in growth cell [53]. *T. pyriformis* has a typical growth curve under normal conditions. This curve is modified under stress conditions. In this work, the results report that FeSO_4_ affects the curve and inhibits protozoan growth. This could be explained by the imbalance between free radicals and antioxidants [54]. The dysregulation of the activities of antioxidants by iron is revealed by the modified activities of antioxidant enzymes (SOD and GPx). This led us to evaluate these last ones and oxidative stress markers (GSH and MDA).

A significant increase in SOD and GPx activities and levels of GSH are observed with iron treatment, proving the induction of oxidative stress by iron. Simultaneously, no change is observed at the MDA level. These results may reflect a defensive reaction against the oxidative effects of metals instead of tolerance to oxidative stress as previously described for cells of mammals [55]. It is well-known that neither H_2_O_2_ nor superoxide are very toxic; however, in the presence of metal, these species are transformed into radicals’ hydroxyls by the Haber–Weiss reaction or the Fenton reaction [56]. The toxic effects of iron on *T. pyriformis* may result in its ability to disrupt mitochondrial functions and cell division, leading to cell death. In addition, antioxidant enzymes are considered as the first line protection mechanism to prevent and reduce oxidative stress [57]. The increased activity of these two enzymes can protect the cells from the production of the oxidative stress marker MDA [58], which explains its normal level even in the presence of iron. In this particular context, treatments with AO and OO show a reduction in oxidative stress compared to the stressed group, as evidenced by the maintenance of normal activities in the combined treatment groups. In the normal case of oxidative stress, ROS oxidizes the main residues of Keap1 cysteine that cause conformational changes and the inability to bind Nrf2 (signaling pathway) [59]. The latter is then transferred to the nucleus [60] where it heterodimerizes with a small Maf protein [61] where it targets the antioxidant response element (ARE) [62]. Once activated, ARE subsequently triggers gene expression such as GPx [63] and SOD [60]. In our case, this main Nrf2 plays a key role in the regulation of gene expression coding for antioxidant enzymes, especially GPx and SOD. The antioxidant characteristics of AO and OO observed on *T. pyriformis* cells reveal that the iron-induced ROS imbalance may be neutralized, which is probably related to its high content of polyphenols, tocopherols, and sterols [64]*,* which allow the cell to develop a compensatory mechanism to cope with oxidative stress instead of borrowing the normal mechanism that usually results in increased activity of these enzymes.

The antioxidant power of both oils is well described in this study using chemical tests and in vitro evaluation. In the same logic, we wanted to study in vivo the preventive effect against oxidative stress. Increased or decreased activity of antioxidant enzymes in various organs reflects an adaptation of cells to oxidative stress induced by iron overload by ROS formation. As we know, oxidative stress is a consequence of the imbalance between the production of free radicals and antioxidant defenses [65]. Many metals can induce oxidative stress as a result of ROS formation [7]. These metals include iron, which is an essential component of living cells and human health. However, an excessive amount of iron leads to overload that could cause various diseases such as chronic liver damage [50]*,* because the liver is the organ responsible for iron storage and regulation [51]. Our results show a significant decrease in GPx and SOD activities. These changes in enzyme activities are concomitant with an increased level of MDA. On the other hand, the GSH content in the liver of mice loaded with iron is not affected by iron treatment. In our case, it seems that hepatic cells do not use the normal line of defense (Nrf2) to cope with oxidative stress, which normally results in increased activity of stress enzymes. This can be explained by the weakening of antioxidant functions by ROS, which is translated by altering the activity of SOD and GPx and increasing the level of MDA. In other words, the antioxidant defense system in the liver was insufficient to offer complete protection against iron-induced damage. The liver reaction to toxic iron is an increase in lipid peroxidation and a decrease in SOD and GPx activities. However, treatment with both oils may increase the antioxidant defense in the liver by limiting the MDA content and simultaneously improving GPx and SOD activities, thus, decreasing lipid peroxidation. The equilibrium resulting from lipid peroxidation in the liver then allows the liver’s antioxidant defense system to effectively protect the organ damage caused by iron overload. In our case, the protection by AO and OO is due to the activation of the Nrf2 pathway by the compounds contained in both oils. As described above, this activation triggers the expression of enzymes of stress and, subsequently, increases enzyme activity.

Oxidative stress is involved in several brain diseases due to its sensitivity to the damages mediated by the ROS [66]. In our study, we show that there is no significant difference in brain activity of GPx and SOD in iron-treated mice. This normal GPx activity is probably related to the non-production of their H_2_O_2_ substrate normally produced by SOD, which also did not have any change. Intriguingly, the treatment by AO or OO shows a decrease in GPx activity, which is conserved just in OO and iron treatment. On the other hand, the concentration of MDA in the brain is not affected by iron treatment in this study, suggesting that there is no effect on the MDA content in the brain. Nevertheless, the marker of GSH oxidative stress is significantly decreased in the brain, which could be related to a decline in microglial cells. These phagocyte resident cells regulate brain homeostasis [67]. As described in the literature, the decrease in GSH levels is the first indicator of oxidative stress in relation to Parkinson’s disease [68]. Similarly, Parkinsonians have high levels of iron [69]. This opens up new perspectives for research in this field. To remedy the harmful effects of iron, we found that the supplementation of oils could counteract them in a way that normalized the level of GSH. In addition to their rich fatty acids, tocopherols and sterols, AO and OO are also rich in polyphenols (vanillic acid, syringic acid, acid ferulic, and tyrosol), with a predominance of ferulic acid in AO and tyrosol in OO. Polyphenols are known as regulators of the Nrf2 signaling pathway in triggering the expression of stress enzymes as described above. It appears that these compounds strengthen the antioxidant defenses of cells by induction of the synthesis of GSH, since even in relatively low concentrations, the polyphenols stimulate gene transcription to the synthesis of GSH in cells [70]. In conclusion, it seems that two oils succeed in strengthening neuronal cells to neutralize the imbalance in iron-induced ROS.

Not only the brain is sensitive to ROS, but also processes involved in the development of many kidney pathologies [32]. Contrary to the results of the brain, the evaluation of oxidative stress shows that there is a significant decrease in GPx and an increase in SOD activities in the kidneys of iron-overloaded mice. The increase in SOD activity is likely due to the activation of the Nrf2 signaling pathway by the ROS generated by the iron stressor. This activation is translated by the expression of the SOD stress enzyme and, subsequently, the increase in its activity. However, the decrease in GPx activity shows that H_2_O_2_ produced by SOD is not taken by GPx to decompose it. This H_2_O_2_ not neutralized can strongly cause cell damage at inappropriate concentrations, causing cell death [71]. In addition, iron treatment does not affected MDA and GSH concentrations in the kidneys in this study. This explains the stability of levels of MDA and GSH in the kidneys. Regardless of these negative effects of iron, AO and OO have successfully counteracted and neutralized them. In addition to that, both oils normalize the SOD and GPx activities, which is probably due to their high content of tocopherols, polyphenols, and phytosterols [72]. Most of these compounds can act in two ways: the first is the activation of the transcription factor Nrf2 [73]*,* and the second is the ability of these compounds to blunt oxidative damage by free radical scavenging mechanisms [74] that are proven by their antioxidant activities (DPPH and FRAP). Oleic fatty acids and 10-oxo-trans-11-octadecoic (derived from linoleic acid), which are abundant in AO and OO, can also provide a protective environment against increased oxidative stress [75,76].

Iron as an oxidizing agent affects all three organs tested at different levels. The liver comes in first followed by the kidneys and the brain in third place. On the other hand, oils moderate oxidative stress by returning stress to a normal level. All our results provide encouraging data on the effects of AO and OO on oxidative stress.

## 4. Materials and Methods

### 4.1. Chemicals and Reagents

All chemicals were purchased from Sigma Aldrich unless otherwise stated.

### 4.2. Origin, Extraction, and Composition of Oils

Dietary argan oil (*Argania spinosa*) was provided by the company EFAS (Agadir, Morocco), and olive oil from the modern cooperative AZZABA (Sefrou, Morocco) in 2018. Argan oil extraction was performed in five different steps: pulping the fruit, crushing the hull, and roasting the almond were performed manually, and grinding roasted almonds was performed by a mechanical press. The olive oil was extracted mechanically. The olive oil was simply extracted by crushing fruits and extracting the juice. The oils were stored at 4 °C in darkness until analysis and used between 1–4 months after purchase. The quality indices and the composition of AO and OO were performed by EFAS company and AZZABA cooperative, respectively. The results of both oils’ composition and quality indices are listed in Table 2 and Table 3, respectively (Supplementary Materials).

The chemical extraction of phenolic compounds from each oil was determined according to the procedure of [47]. Briefly, 2 g of the oil was dissolved in 1 mL of n-hexane and the solution was shaken, followed by a liquid–liquid extraction using 2 mL of methanol/water (80: 20 *v*/*v*). The mixture was centrifuged at 2800× *g* for 5 min, the extraction was repeated in the lipophilic fraction and the methanolic fractions were combined.

**Table 2:** Fatty acid composition (%) of argan oil (*Argania spinosa*) and olive oil.

Fatty Acids (%)	Argan Oil	Olive Oil
C14:0 (myristic acid)	0.1	0.0
C15:0 (pentadecanoic acid)	<0.1	ND ^a^
C16:0 (palmitic acid)	12.6	9.7
C17:0 (margaric acid)	0.1	0.0
C17:1 (heptadecenoic acid)	<0.1	0.1
C18:0 (stearic acid)	5.8	2.6
C18:1 (oleic acid)	46.3	73.6
C18:2 (linoleic acid)	34.0	11.3
C18:3 (linolenic acid)	0.1	1.0
C20:0 (arachidic acid)	0.3	0.3
C20:1 (gadoleic acid)	0.3	0.4
C22:0 (behenic acid)	0.1	0.1
C24:0 (lignoceric acid)	ND ^a^	0.2
C18:1t	ND ^a^	0.02
C18:2t + C18:3t	ND ^a^	0.04

^a^: Not detected, t: trans.

**Table 3:** Acidity, peroxide value, specific extinction coefficient (at 232 nm and 270 nm wavelength), ΔK, moisture, and volatile matter of argan oil (*Argania spinosa*) and olive oil.

	Argan Oil	Olive Oil
Acidity (% as oleic acid)	0.22	0.28
Peroxide value (meq O_2_/kg oil)	2.1	3.2
K232	ND ^a^	1.71
K270	0.13	0.15
ΔK	0.003	0.00
Moisture and volatile matter (%)	0.02	0.13

^a^: Not detected.

### 4.3. Total Polyphenol Contents

The total phenolic content (TPC) of the methanolic fraction of oil was estimated by Folin–Ciocalteu [77]. Briefly, 100 µL of the methanolic fraction was mixed with 900 µL of Folin–Ciocalteu reagent (diluted 1:10 water). After 5 min, 750 µL of sodium carbonate (6% *w*/*v*) was added. The solution was vigorously shaken and allowed to stand for 90 min. The absorbance was measured at 700 nm using a spectrophotometer. The extraction solvent was used for blank controls instead of a methanolic fraction of oil. All values were expressed as mg of gallic acid equivalent per kg of oil (GAE/kg oil).

### 4.4. Determination of Chlorophyll and Carotenoid Contents

The chlorophyll and carotenoid contents were determined by dissolving 1.5 g of oil in a final volume of 5 mL of cyclohexane [78]. The amounts of carotenoid and chlorophyll were calculated by measuring the absorbance at 470 nm and 670 nm, respectively, according to the following equations:

Carotenoid (mg/Kg) = (A_470_ × 10^6^)/(E_1_ × 100)

Chlorophyll (mg/Kg) = (A_670_ × 10^6^)/(E_2_ × 100)

A is the absorption at 470 or 670 nm; E_1_ is the extinction coefficient of lutein = 2000; E_2_ is the extinction coefficient of pheophytine 613.

### 4.5. Antioxidant Activities

#### 4.5.1. FRAP Assay

The reducing power of the methanolic extract was determined according to the procedure described by [79]. A total of 500 µL of the methanolic fraction of oil was mixed with 1.25 mL phosphate buffer (0.2 M, pH 6.6) and 1.25 mL of potassium ferric cyanide (K_3_Fe(CN)_6_) (1%). The mixture was incubated at 50 °C for 20 min. To stop the reaction, 1.25 mL trichloroacetic acid (10%) was added. The mixture was centrifugated at 1000× *g* for 10 min. Then, 1.25 mL of the supernatant was mixed with 1.25 mL of distilled water and 500 µL of the aqueous of FeCl_3_ (0.1%). The absorbance was measured at 700 nm by a spectrophotometer. All values were expressed as mmol of Trolox equivalent per kg of oil (TE/kg oil).

#### 4.5.2. DPPH Assay

The antiradical activity of ethyl acetate extract of oil was estimated according to [80] with slight modifications. Briefly, 1 mL of oil fraction (10%) was added to 4 mL of DPPH (0.1 mM) solution, the mixture was vortexed and placed in the dark for 30 min. The absorbance was measured at 515 nm by a spectrophotometer. All values were expressed as mmol of Trolox equivalent per kg of oil (TE/kg oil).

### 4.6. Culture and Treatments

#### 4.6.1. Culture Medium

*T. pyriformis* was kindly provided by the Laboratory of Physiology and Molecular Genetics, Department of Biology, Faculty of Sciences Ain Chock, University Hassan II-Ain Chock, Casablanca, Morocco. The culture medium (PPYG) of *T. pyriformis* contained 0.4% protease peptone, 0.2% yeast extract, and 1% glucose [81]. The culture medium and consumables were sterilized in high-pressure steam at 120 °C before use. During the growth, the cells entered the exponential phase with a high density, and then a pre-stationary phase of growth followed by a stationary phase [82]. In our study, the cell cultures were always performed in the exponential phase in a new fresh medium before each treatment with the tested products. The assays always departed from initial densities of about at least 10^4^ cells per mL.

#### 4.6.2. Iron Treatment

The *T. pyriformis* growth curve was produced to obtain adequate cell densities for the treatment with different iron sulfate concentrations. Cell density was determined using a hemocytometer with an optical microscope. After 24 h of culture, the medium was mixed in triplicate with different iron sulfate concentrations (from 0.3 to 4 mM of FeSO_4_). Sterile distilled water was set as a control. Cells were kept in the incubator at 28 °C for an additional 24 h and the IC50 was determined. The FeSO_4_ was prepared in sterile distilled water just before use.

#### 4.6.3. Argan Oil and Olive Oil Treatment

To treat *T. pyriformis* with AO or OO., a stock solution of argan and olive oil was prepared at 10% (*v*/*v*) in absolute ethanol. For cell treatment, the final concentration in the medium with oil and ethanol was 0.1% and 0.9%, respectively [17]. After 2 h of incubation of cells in the presence of AO or OO, the cells were treated by the stress agent at the IC50 concentration (Table 4).

### 4.7. Animals and Experimental Design

Male Swiss OF1 mice between 12 and 16 weeks of age were purchased from IFFA CREDO in Casablanca, Morocco. They were acclimatized to our laboratory conditions for 10 days in a pathogen-free environment at 22 ± 2 °C and a constant light–dark cycle (12 h–12 h). They were housed in six groups (at least 3 mice/group) and fed with standard lab food and water ad libitum. Experiments were carried out following the Institutional Animal Ethics Committee of Hassan First University of Settat, Morocco, and according to the National Institutes of Health Guide for the Care and Use of Laboratory Animals (NIH publication no. 85-23, revised 1985). All efforts were made to minimize animal suffering and the number of animals used. Argan oil used in this study was obtained from Agadir City, Morocco. The concentration of iron used in our study was calculated to exceed the normal concentration of this chemical found in drinking water according to the World Health Organization (WHO), which is less than 0.3 mg/liter [83]. Before treatment (Table 5), the chow was kept under a hood to evaporate the acetone, which served as the diluent for the oils. Six groups of mice were distributed for 28 days as follows: group I: a standard chow (control) supplemented with acetone. Group II: a standard chow supplemented with 6% of argan oil (AO) solubilized in acetone. Group III: a standard chow supplemented with 6% of olive oil (OO) solubilized in acetone. Group IV: the reference drug Tardyferon (iron sulfate 3.5 mg Fe^2+^/liter) was dissolved in drinking water. Group V: the animals received iron and AO. Group VI: the animals received iron and OO. During 28 days, mice were weighed weekly using an electronic balance. At the end of the treatment period, liver, kidneys, and brain tissues were frozen in a dry ice bath and stored at −80 °C for further analysis.

### 4.8. Tetrahymena Pyriformis

The cells were harvested by centrifugation at 6000× *g* for 10 min and suspended in 50 mM phosphate buffer, pH 7.4. The cells were then disrupted on ice with a homogenizer model Heidolph DIAX 600 and sonicated on ice using Sonifier (3 cycles, 30 s). The supernatant (soluble protein fraction) obtained after centrifugation at 15,000× *g* for 45 min at 4 °C was considered as the crude cell-free extract and small aliquots were stored at −80 °C until further analyses.

### 4.9. Liver, Brain, and Kidney

A 10% (*w*/*v*) liver, kidney, and brain homogenates were prepared in 50 mM phosphate buffer (KH_2_PO_4_; K_2_HPO_4_; pH 7.4) using a potter. The supernatant (soluble protein fraction) obtained after centrifugation at 3000× *g* for 10 min at 4 °C was considered as the crude cell-free extract and small aliquots were stored at −20 °C until further analyses.

### 4.10. Animals and Experimental Design

The homogenate protein content was measured, according to the procedure described by [84] using bovine serum albumin as a standard.

### 4.11. Oxidative Stress Markers and Antioxidants

#### 4.11.1. Lipid Peroxidation

The level of lipid peroxidation was evaluated by measuring the content of malondialdehyde, a product of lipid peroxidation. MDA was determined by the thiobarbituric acid (TBA) reaction [85]. The principle of the MDA assay is based on the reaction of two molecules of TBA with one molecule of MDA forming a colored MDA–TBA2 adduct that absorbs strongly at 532 nm. The n-butanol is used to remove any remaining hemoglobin in the sample that could cause a false positive due to hemoglobin’s absorbance at 540 nm, which is close to the adduct absorbance. A total of 500 µL of homogenate was added to 500 µL of trichloroacetic acid (20%) and 1 mL of thiobarbituric acid (0.67%). The mixture was heated at 100 °C for 15 min and then quickly cooled in an ice bath; 4 mL of n-butanol was added. After centrifuging at 3000× *g* for 15 min, the absorbance of the supernatant at 532 nm was read. The MDA level was expressed as nanomoles of MDA per milligram of protein.

#### 4.11.2. Glutathione

The level of GSH was measured by the method of [86]. The principle of the GSH assay is based on the oxidation of GSH present in the sample by the sulfhydryl reagent 5,5′-dithiol-bis (2-nitrobenzoic acid) (DTNB) to form the yellow-colored derivative 5′-thio-2-nitrobenzoic acid (TNB). The reaction mixture contained 200 µL TCA (5%) and 400 µL homogenate. After centrifuging at 12,000× *g* for 10 min, 50 µL of supernatant was added to 100 μL of DTNB (6 mM) and 850 μL of 50 mM phosphate buffer, pH 8. The absorbance was measured spectrophotometrically at 412 nm after 5 min.

### 4.12. Enzymatic Activity Measurements

#### 4.12.1. *Glutathione Peroxidase*

The GPx activity was assayed as described by [87]. The principle of the GPx assay is based on the ability of GPx to catalyze the oxidation of GSH by the sulfhydryl reagent 5,5′-dithiol-bis (2-nitrobenzoic acid) (DTNB) to form the yellow-colored derivative 5′-thio-2-nitrobenzoic acid (TNB). The rate of TBN formation, which is directly proportional to the GPx activity, can be measured spectrophotometrically at 412 nm. The reaction mixture that contained 200 µL of the homogenate was mixed with 400 µL potassium phosphate buffer (0.4 mM, pH 7.0), 200 µL GSH (2 mM), 200 µL of EDTA (0.8 mM), 100 µL sodium azide (10 mM), and 100 µL of H_2_O_2_ (2.5 mM). After 10 min of incubation at 37 °C, the reaction was stopped by adding 500 µL of TCA (10%) and then centrifuged at 2000× *g* for 5 min. Then, 2.5 mL of sodium phosphate tribasic (0.1M) and 1 mL of DTNB buffer (0.04%) were added to the supernatant. The absorbance was measured spectrophotometrically at 412 nm after 2 min. The specific activity of GPx was expressed as micromoles of GSH per minute per milligram of protein.

#### 4.12.2. Superoxide Dismutase

The SOD activity was assayed as described by [88]. The principle of the SOD assay is based on the illumination of riboflavin solution in the presence of EDTA, which causes a reduction in the flavin. It then re-oxidizes and simultaneously reduces oxygen to O^2−^, which is allowed to react with a detector molecule NBT, reducing the NBT to a formazan blue. The SOD in the sample inhibits the formazan production, resulting in a decrease in the color intensity of the formed blue precipitate that absorbs strongly at 560 nm. The 3 mL reaction mixture contained 50 mM phosphate buffer, pH 8 0.025% triton x-100, 0.1 mM EDTA, 12 mM L-Methionine, 75 mM NBT, homogenate, and 2 µM riboflavin. The tubes were shaken and placed 30 cm below a light bank consisting of a 15 W fluorescent lamp for 10 min. The reaction was stopped by switching off the light and the absorbance was measured spectrophotometrically at 560 nm.

### 4.13. Statistical Analysis

All results were expressed as mean ± standard deviation. The statistical analysis to compare the two groups was performed with an unpaired, two-tailed, Student *t*-test; a *p*-value of 0.05 or less was deemed to be statistically significant.

## 5. Conclusions

In the present study, we evaluated the potential protective effect of argan oil and olive oil against oxidative stress induced by ferrous sulfate in vitro in the protozoan *Tetrahymena pyriformis* and in vivo in mice. Our data show the antioxidant effects of argan oil and olive oil against iron-induced oxidative stress: in vivo in the liver, kidney, and heart; and in vitro in the *Tetrahymena pyriformis*. These results reveal that the iron-induced ROS imbalance can be scavenged by AO and OO, which is probably related to their particular composition, known for their antioxidant activities.

## Figures and Tables

**Table 4:** Conditions of treatment “In-vitro”.

Groups	Treatment
Group I	Culture medium with the solvent ethanol
Group II	Culture medium (as group I) supplemented with 0.1% of AO solubilized in ethanol
Group III	Culture medium (as group I) supplemented with 0.1% of OO solubilized in ethanol
Group IV	Culture medium (as group I) supplemented with iron at 1.849 mM dose dissolved in water
Group V	Culture medium (as group I) and AO (as group II)
Group VI	Culture medium (as group I) and AO (as group III)

**Table 5:** Conditions of treatment “In-vivo”.

Groups	Treatment
Group I	A standard chow with the solvent acetone
Group II	A standard chow (as group I) supplemented with 6% of AO solubilized in acetone
Group III	A standard chow (as group I) supplemented with 6% of OO solubilized in acetone
Group IV	The reference drug Tardyferon (iron sulphate 3.5 mg^2+^/liter) was dissolved in drinking water
Group V	The animals received iron (as group IV) and AO (as group II)
Group VI	The animals received iron (as group IV) and OO (as group III)

## Data Availability

Data are contained within the article.

## Supplementary Materials

The following supporting information can be downloaded at: https://www.mdpi.com/article/10.3390/molecules28155924/s1.
