# Supplementary material for: Antioxidant Effects of Argan Oil and Olive Oil against Iron-Induced Oxidative Stress: In Vivo and In Vitro Approaches
Source: Molecules. 2023 Aug 7;28(15):5924. doi: 10.3390/molecules28155924 (PMC10420636; doi:10.3390/molecules28155924)
Supplement: Supplementary file 1 [file molecules-28-05924-s001.zip › Composition Report Argan oil (1).pdf]

11

ETABLISSEMENT AUTONOME DE CONTROLE
ET DE COORDINATION DES EXPORTATIONS
LABORATOIRE AGADIR

المملكة المغربية
المؤسسة المستقلة لمراقبة
وتنسيق الصادرات
مختبر أكادير

ESSAIS

Rapport d'analyses

Section Corps Gras

LotissementLn° 13, zone industrielle, Tassila, Agadir, Maroc.Tél., 212.(520)32.20.17. Site web.: http://web2.eacce.org.ma. Email.: eacce@eacce.org.ma

Echantillon : 2017-04408

Dénomination : HUILE ARGANE TORREFIEE
Description : Deux bouteilles en verre de 250ml fermées
Numéro de Prélèvement : 337/17
Demande d'analyse : 1708003769
Producteur : EFAS
Exportateur : EFAS
Fabricant ou conditionneur : EFAS
Destination : UNION EUROPEENNE
Date Fabrication : ---

Date Conditionnement : ---
Section : Corps Gras.
Marque : ---
Lot : A01/17
Client : Délégation Agadir PV
Emballage : BOUTEILLE EN VERRE
Date de prélèvement : 03-08-2017
Date d'entrée : 04-08-2017
Date de fin d'analyse : 12-08-2017

	LQ	Résultat	Unité	Incertitude	Normes	Méthode
Absorbance dans l'Ultraviolet (K1% 1cm)*						NF EN ISO 3656, COI T.20 Doc n°19
Absorbance (K) à 270nm *		0,13				
Delta K *		0,003				
Composition en acides gras*						NF EN ISO 12966-2(Méthode transméthylation en conditions de catalyse alcalines),NF EN ISO 12966-4
ACIDE MYRISTIQUE (C14:0) *		0,1	%			
ACIDE PENTADECANOIQUE (C15:0) *		<0,1	%			
ACIDE PALMITIQUE (C16:0) *		12,6	%			
ACIDE PALMITOLEIQUE (C16:1) *		0,2	%			
ACIDE MARGARIQUE (C17:0) *		0,1	%			
ACIDE HEPTADECENOIQUE (C17:1) *		<0,1	%			
ACIDE STEARIQUE (C18:0) *		5,8	%			
ACIDE OLEIQUE (C18:1) *		46,3	%			
ACIDE LINOLEIQUE (C18:2) *		34,0	%			
ACIDE LINOLENIQUE (C18:3) *		0,1	%			
ACIDE ARACHIDIQUE (C20:0) *		0,3	%			
ACIDE GADOLEIQUE (C20:1) *		0,3	%			
ACIDE BEHENIQUE (C22:0) *		0,1	%			
Acidité libre % exprimée en acide oléique*						NF EN ISO 660 (Méthode au solvant à froid utilisant un indicateur)
ACIDITE LIBRE *		0,22	%			
Teneur en eau et matières volatiles*						NF EN ISO 662 (Méthode B)
Teneur en eau et en matières volatiles (M/M) *		0,02	%			
Indice de peroxyde (méq/O2 actif par Kg)*						ISO 3960
Indice de peroxyde en milléquivalent O2/kg *		2,1	meq/o2 actif par kg			

Ce rapport d'analyse est destiné au client cité ci dessus. Il ne peut être reproduit, sinon en entier, sans autorisation écrite du responsable Laboratoire Agadir

Scanned by CamScanner

Scanné avec CamScanner

ROYAUME DU MAROC
ETABLISSEMENT AUTONOME DE CONTROLE
ET DE COORDINATION DES EXPORTATIONS
LABORATOIRE AGADIR

المملكة المغربية
المؤسسة المستقلة لمراقبة
وتنسيق الصادرات
مختبر أكادير

Rapport d'analyses

Section Corps Gras

Lotissement n° 13, zone industrielle, Tassila, Agadir, Maroc, Tél : 212.(528)32.20.17, Site web : http://web2.eacce.org.ma, Email : eacce@eacce.org.ma

Echantillon : 2017-04408

Conclusion au vu des analyses effectuées :

[illegible]sultats ne se rapportent qu'à l'échantillon soumis à l'essai

Edité le : 12/08/2017

[illegible]ccréditation du COFRAC atteste de la compétence du laboratoire pour les seules déterminations couvertes par l'accréditation qui sont identifiées par le symbole : *

Responsable de la Section
Yassine EL ALEM

Responsable du Laboratoire
Lhoucine BAZZI

Responsable du Laboratoire d'Analyses
Physico-Chimiques AGADIR
Lhoucine BAZZI

Ce rapport d'analyse est destiné au client cité ci-dessus. Il ne peut être reproduit, sinon en entier, sans autorisation écrite du responsable Laboratoire Agadir

Scanned by CamScanner

Scanné avec CamScanner
