# Supplementary material for: Antioxidant Effects of Argan Oil and Olive Oil against Iron-Induced Oxidative Stress: In Vivo and In Vitro Approaches
Source: Molecules. 2023 Aug 7;28(15):5924. doi: 10.3390/molecules28155924 (PMC10420636; doi:10.3390/molecules28155924)
Supplement: Supplementary file 1 [file molecules-28-05924-s001.zip › Composition Report Olive oil.pdf]

ROYAUME DU MAROC
ETABLISSEMENT AUTONOME DE CONTROLE
ET DE COORDINATION DES EXPORTATIONS
LABORATOIRE MEKNES

المملكة المغربية
المؤسسة المستقلة لمراقبة وتنسيق الصادرات
مختبر مكناس

cofrac ESSAIS
Laboratoire d'essai accrédité sous le N° 1-5846 et portée disponible sur : www.cofrac.fr

Rapport d'analyses

Laboratoire d'analyses physicochimiques-Section Corps Gras

Qualipôle, Agropolis de Meknès,Tél : +212 6 18 53 22 43, Site web : http://www.eacce.org.ma, Email : eacce@eacce.org.ma

Echantillon : 2017-07493

INFORMATIONS CLIENT

Demande d'analyse : 1712006367
Dénomination : **HUILE OLIVE EXTRA VIERGE**
Description : DEUX BOUTEILLES DE 250 ml EN PLASTIQUE FERMEES
Numéro de Prélèvement : 204/17F
Date de prélèvement : 21-12-2017
Exportateur : HUILE DE SEFROU
Fabricant ou conditionneur : HUILE DE SEFROU
Producteur : GIE HUILE DE SEFROU
Date Conditionnement : ---
Date Fabrication : ---
Marque : ---
Lot : 2 PEV 2011-2016
Destination : UNION EUROPEENNE
Emballage : .
Client : Délégation Fès

INFORMATIONS LABORATOIRE

Section : Corps Gras..
Date d'entrée : 03-01-2018
Date d'exécution : 03-01-2018
Date de fin d'analyse : 09-01-2018

		LQ	Résultat	Unité	Incertitude	Normes	Méthode
ACIDITE(EN %ACIDE OLEIQUE)*							**NF EN ISO 660**
Acidité (En % Acide Oléique)	*		0,28	%		<= 0,8	
INDICE DE PEROXYDE*							**ISO 3960**
Indice de peroxyde en milliéquivalent O2/kg	*		3,2	meq/o2actif/Kg		<= 20	
EXTINCTION SPECIFIQUE A 232nm							**ISO 3656 /COI/T.20/DOC.N°19**
Extinction Spécifique à 232 nm			1,71			<= 2,50	
EXTINCTION SPECIFIQUE A 270nm							**ISO 3656 /COI/T.20/DOC.N°19**
Extinction Spécifique à 270 nm			0,15			<= 0,22	
DELTA K							**ISO 3656 /COI/T.20/DOC.N°19**
Delta K			0,00			<= 0,01	
TENEUR EN EAU ET EN MATIERES VOLATILES*							**NF EN ISO 662**
Teneur en eau et en matières volatiles (M/M)	*		0,13	%		<= 0,2	
Analyse des esters methyliques des acides gras*							**ISO 12966-2 ET NF EN ISO 12966-4**
ACIDE MYRISTIQUE (C14:0)	*		0,0	%		<= 0,03	
ACIDE PALMITIQUE (C16:0)	*		9,7	%		>= 7,50 <= 20,00	
ACIDE PALMITOLEIQUE (C16:1)	*		0,5	%		>= 0,30 <= 3,50	
ACIDE MARGARIQUE (C17:0)	*		0,0	%		<= 0,40	

Ce rapport d'analyse est destiné au client cité ci-dessus. Il ne peut être reproduit, sinon en entier, sans autorisation écrite du responsable Laboratoire Meknes

ROYAUME DU MAROC
ETABLISSEMENT AUTONOME DE CONTROLE
ET DE COORDINATION DES EXPORTATIONS
LABORATOIRE MEKNES

EACCE

المملكة المغربية
المؤسسة المستقلة لمراقبة
وتنسيق الصادرات
مختبر مكناس

cofrac ESSAIS
Laboratoire d'essai accrédité sous le N° **1-5846** et portée disponible sur : www.cofrac.fr

Rapport d’analyses

Laboratoire d'analyses physicochimiques-Section Corps Gras

Qualipôle, Agropolis de Meknès,Tél : +212 6 18 53 22 43, Site web : http://www.eacce.org.ma, Email : eacce@eacce.org.ma

Echantillon : 2017-07493

ACIDE HEPTADECENOIQUE (C17:1) *		0,1	%		<= 0,60	
ACIDE STEARIQUE (C18:0) *		2,6	%		>= 0,50 <= 5,00	
ACIDE OLEIQUE (C18:1) *		73,6	%		>= 55,00 <= 83,00	
ACIDE LINOLEIQUE (C18:2) *		11,3	%		>= 2,50 <= 21,00	
ACIDE LINOLENIQUE (C18:3) *		1,0	%		<= 1,00	
ACIDE ARACHIDIQUE (C20:0) *		0,3	%		<= 0,60	
ACIDE GADOLEIQUE (C20:1) *		0,4	%		<= 0,50	
ACIDE BEHENIQUE (C22:0) *		0,1	%		<= 0,20	
ACIDE LIGNOCERIQUE (C24:0) *		0,2	%		<= 0,20	
AUTRE *		0,1	%			
Analyse des esters methyliques des acides gras trans						**ISO 12966-2 ET NF EN ISO 12966-4**
C 18 : 1 TRANS		0,02	%		<= 0,05	
C18:2 Trans + C18:3 Trans en %		0,04	%		<= 0,05	

Rapport d’analyses

Laboratoire d'analyses physicochimiques-Section Corps Gras

Qualipôle, Agropolis de Meknès,Tél : +212 6 18 53 22 43, Site web : http://www.eacce.org.ma, Email : eacce@eacce.org.ma

Echantillon : 2017-07493

Conclusion au vu des analyses effectuées : ECHANTILLON CONFORME AUX NORMES EN VIGUEUR DU CONSEIL OLEICOLE INTERNATIONAL SANS TENIR COMPTE DE L'INCERTITUDE ASSOCIÉE AUX RÉSULTATS.

Edité le : 08/06/2023

Les résultats ne se rapportent qu’à l’échantillon soumis à l'essai

L’accréditation du COFRAC atteste de la compétence du laboratoire pour les seules déterminations couvertes par l'accréditation qui sont identifiées par le symbole : *

Responsable de la Section

Sofia EL MARJANY

Responsable du Laboratoire

Ahmed SMIHROU
